# Characterization of the mitochondrial genome and phylogenetic position of the Varied Solitaire *Myadestes coloratus* (Passeriformes: Turdidae)

**DOI:** 10.1080/23802359.2025.2549750

**Published:** 2025-08-25

**Authors:** Jorge L. Garzon, Celestino Aguilar, Kristin Saltonstall, Carlos F. Arias, Catherine B. Viverette, Luis F. De León

**Affiliations:** ^a^ Integrative Life Sciences Doctoral Program, Virginia Commonwealth University, VA, USA; ^b^ Smithsonian Tropical Research Institute, Ancon, Panama; ^c^ Department of Genomics and Proteomics, Gorgas Memorial Institute for Health Studies, Panama, Republic of Panama; ^d^ Departamento de Microbiologia Humana, Facultad de Medicina, Universidad de Panamá, Panama, Republic of Panama; ^e^ VCU Rice Rivers Center, Virginia Commonwealth University, Richmond, Virginia, USA; ^f^ Department of Biology, University of Massachusetts Boston, Boston, MA, USA

**Keywords:** Mitogenome, neotropical birds, Panama, endemic, Solitario Variado

## Abstract

The Varied Solitaire (*Myadestes coloratus* Nelson, 1912) is a near-threatened bird species, endemic to the highlands of eastern Panama and the Colombian border. In this study, we report the complete mitochondrial genome of *M. coloratus*. The mitogenome exhibits a typical avian structure with a slight AT bias and a control region. Phylogenetic analysis confirms the close relationship between *M. coloratus* and other thrush (Turdidae) species, including the extinct *M. myadestinus*. This data is crucial for future research on the evolutionary history and population genetics of *M. Coloratus*. These findings are particularly relevant as the species is increasingly threatened by human expansion.

## Introduction

*Myadestes coloratus* Nelson, 1912, commonly known as the Varied Solitaire, is an endemic thrush (Turdidae) found in the highlands between eastern Panama and the border with Colombia (Ridgely and Gwynne 1989; Collar 2020). This region is crucial for the conservation of several endemic bird species and subspecies, which are restricted to forests above 600 m in elevation (Renjifo et al. 2017; Aguilar et al. 2025; Garzon and De León 2025). Unfortunately, these habitats are increasingly threatened by deforestation and human expansion, likely leading to the decline of several animal populations in the region (Batista et al. 2020). Consequently, *M. coloratus* has been classified as near-threatened by the IUCN Red List (IUCN 2021).

However, limited genetic information on *M. coloratus* is currently available, likely due to its restricted range and remote habitat (Garzon 2021). While one complete mitochondrial genome exists for the genus, from the now-extinct Hawaiian *M. myadestinus* (IUCN 2016; Anmarkrud and Lifjeld 2017), no complete mitogenome has been reported for any Neotropical species of *Myadestes*. For *M. coloratus*, only partial sequences of ATP8/6, COX1, Cytb, and ND2 genes are available (Miller et al. 2007). This paucity of data limits understanding of the species’ evolutionary history and conservation status (De León et al. 2023; Aguilar et al. 2025). Here, we sequence and annotate the complete mitochondrial genome of *M. coloratus*, to support future research on its phylogenetic history, population structure, as well as conservation efforts.

## Materials and methods

**Figure 1.**
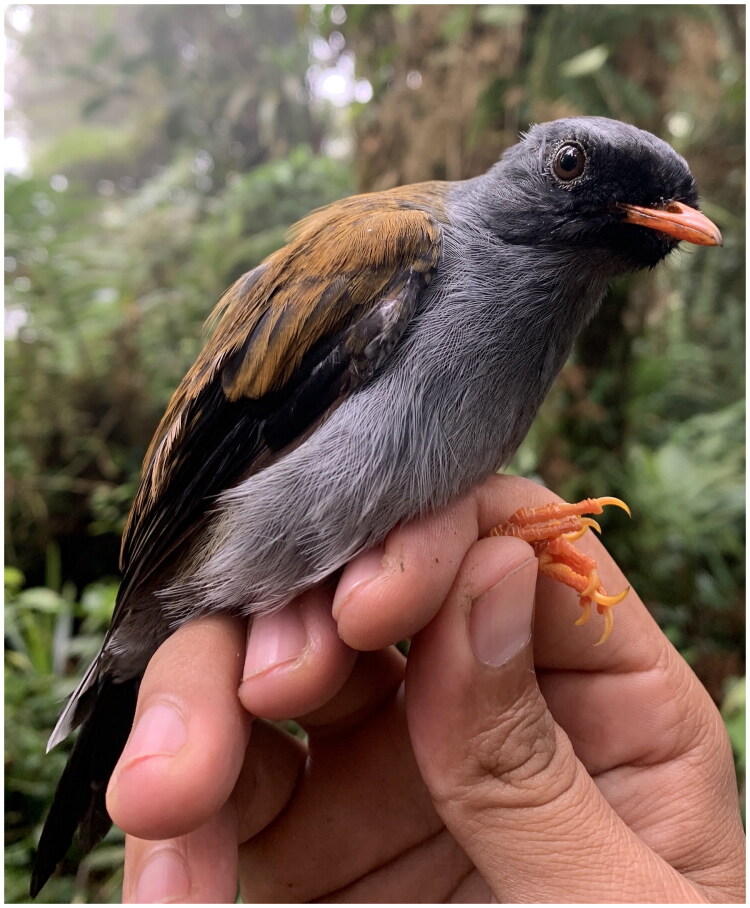
Reference photograph of Varied Solitaire (*M. coloratus*). The species is characterized by its black facial mask extending from the bill to mid-eye level, dark ashy gray head and underparts, and tawny-rusty rufous upper parts with olivaceous tones. Its beak and legs are orange (Ridgely and Gwyne 1989). Photo taken by Jorge L. Garzón in Cerro Chucantí, Darién, Panamá (8.80147 N, −78.46095 W).

DNA was extracted from a tissue of a deceased individual of *M. coloratus* previously collected in Serranía de Majé, Cerro Chucantí (8.811069 N, −78.457869 W) on 26 January 2006, under the permit DNAPVS-01-2006 from the Authority of the Environment (ANAM). The specimen was identified as *M. coloratus* based in diagnostic morphological features described in A Guide to the Birds of Panama: with Costa Rica, Nicaragua, and Honduras (Ridgely and Gwynne 1989), including its distinctive black facial mask, ashy-gray underparts and tawny-rufous upper part with olivaceus (Figure 1). The orange beak and legs supported the identification. The individual was an adult, confirmed by its fully developed definitive plumage. The voucher is deposited at the Smithsonian Tropical Research Institute Bird-Tissue Collection, Naos Island, Panama (María F. Castillo is the contact person: castillomf@si.edu) under the ID number GMS1933.

The mitochondrial genome was assembled from reads obtained from hybrid capture libraries originally prepared for ultra-conserved element loci (UCEs: (Faircloth et al. 2012). In brief, total DNA was extracted using a DNeasy Blood & Tissue Kit (Qiagen, Valencia, California, USA) following the manufacturer’s protocol, with DNA quality evaluated *via* nanodrop and Qubit 3 fluorometry. Libraries were paired-end sequenced (2 × 150 bp) on an Illumina HiSeq (Illumina, San Diego, CA, USA) at the Arbor BioScience laboratories (Michigan, USA). After quality control and trimming using FastQC and Illumiprocessor (Faircloth 2013), *de novo* assembly was performed using Unicycler v. 0.5.0 (Wick et al. 2017). Through BLAST (Altschul et al. 1990) analysis, we identified one large contig sequence that matched the mitochondrial genome *M. coloratus*. The sequence was annotated using MitoAnnotator (Iwasaki et al. 2013) and MITOS2 (Bernt et al. 2013), with final curation in Geneious Prime v. 2024.2 (Kearse et al. 2012).

To establish the phylogenetic framework of the reported mitogenome, we downloaded published mitogenomes of Turdidae from NCBI. We also used one published mitogenome of *Passer montanus* as an outgroup. The nucleotide sequences of the 13 PCGs from each mitogenome were extracted manually using Geneious, aligned using MAFFT (Katoh and Standley 2013), and concatenated using Geneious Prime 2024. Phylogenetic analysis with maximum likelihood (ML) was then performed using IQ-TREE2 (Minh et al. 2020) under the best-fit model GTR+I + G4 determined using ModelFinder (Kalyaanamoorthy et al. 2017) and 1000 bootstrap replicates. Finally, we used FigTree 1.4.4 (Rambaut and Drummond 2012) to visualize the resulting phylogenetic tree.

## Results

**Figure 2.**
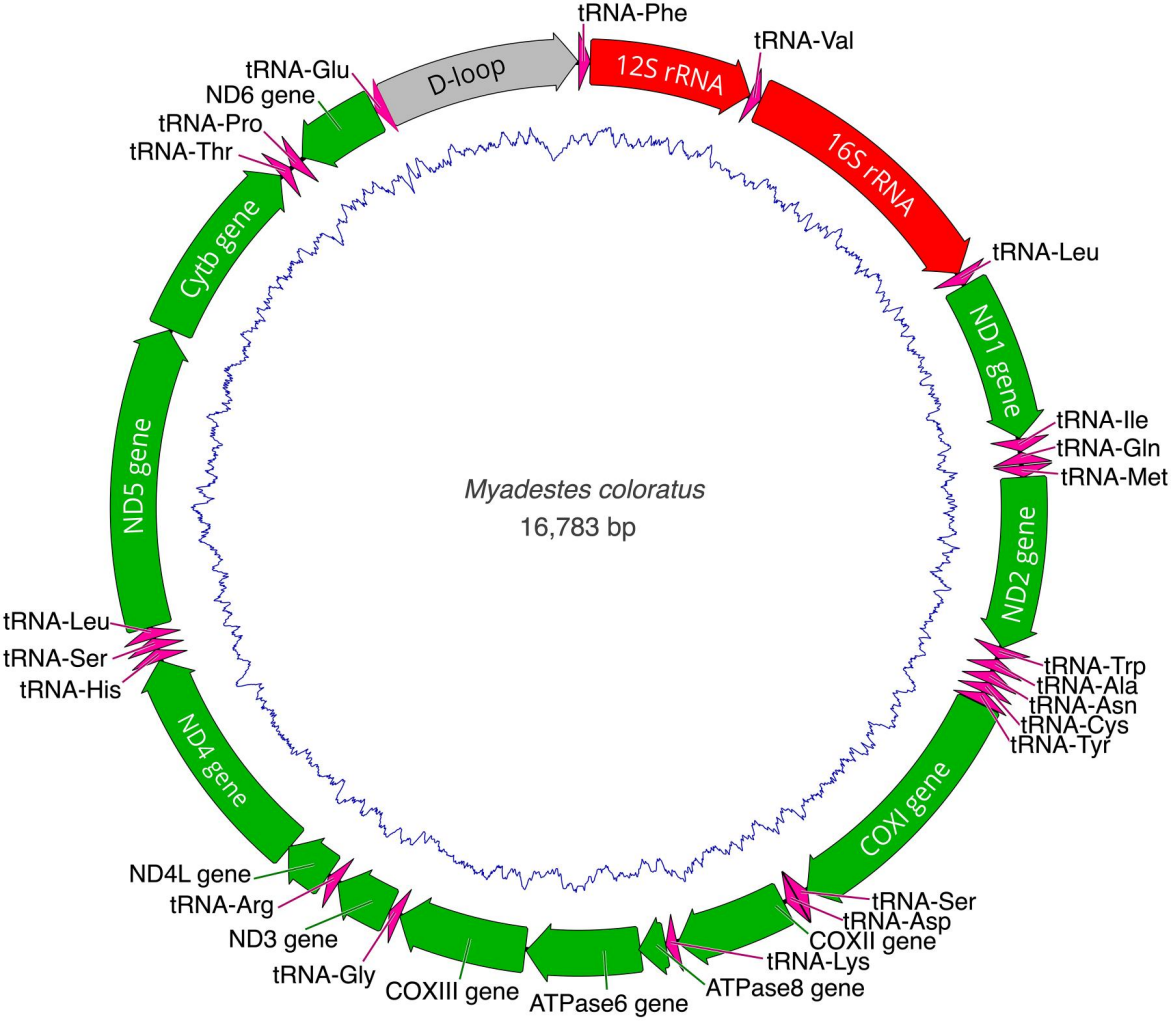
Assembly and annotation of the *M. coloratus* mitochondrial genome. Green-colored bars represent coding sequences for protein-coding genes, red bars represent rRNA genes, magenta bars represent tRNA genes, and gray bars represent control regions. The direction of transcription is indicated by the orientation of the gene arrows. The inner blue line represents GC content across the mitogenome. The mitogenome assembly was visualized using Geneious Prime v. 2023.2.1 software.

The complete mitochondrial genome of *M. coloratus* (Figure 2) was recovered for individual GMS1933, and the consensus sequence length was 16,783 bp with a mean coverage of 19×. The depth of the mitogenome coverage map is shown in the Supplementary Figure S1. The complete genome sequence had been deposited in GenBank under the accession number PQ872305. The base composition of mitochondrial DNA (mtDNA) showed that the percentage of A + T (53.7%) was slightly higher than G + C (46.3%). The complete mtDNA sequence contained 13 protein-coding genes (PCGs), 22 tRNA genes, two rRNA genes (12S rRNA and 16S rRNA), and a control region. The 13 PCGs encoded ND1, ND2, COX1, COX2, ATP8, ATP6, COX3, ND3, ND4L, ND4, ND5, CYTB, and ND6, respectively. Of the 13 PCGs, 12 utilized ATG as the start codon, whereas COX1 started with GTG. Six PCGs ended with TAA (CYTB, COX2, ATP8, ATP6, ND3, and ND4L), two ended with AGG (ND1 and COX1), one with AGA (ND5), one with TAG (ND6), and the other three ended with incomplete TA-/T– (ND2, ND4, and COXIII) stop codons. The 22 tRNA genes ranged in size from 66 bp encoding tRNA-Ser1 to 76 bp encoding tRNA-Ser2. The 12S and 16S rRNA genes were 983 bp and 1600 bp, respectively. The non-coding control region (CR) was located between tRNA-Glu and tRNA-Phe (Figure 2).

**Figure 3.**
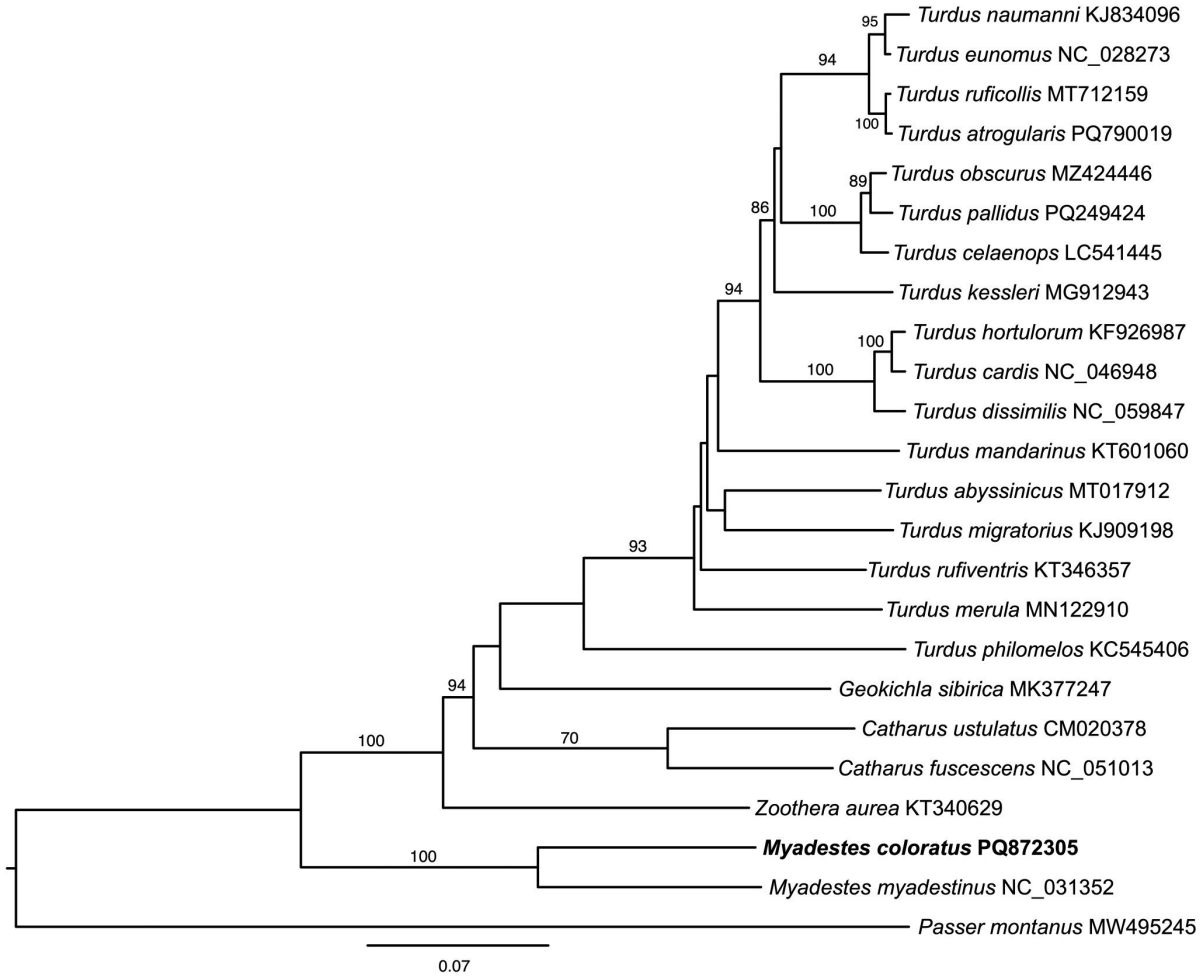
Phylogenetic position of *Myadestes coloratu*s within the family Turdidae based on complete mitochondrial genomes. The phylogenetic tree was based on maximum likelihood analysis of 13 PCGs and included 22 species of Turdidae for which mitochondrial genomes were available. ML bootstrap support values ≥70% are shown along the nodes. The GenBank accession numbers are displayed after the species name. The following sequences were used: *Turdus naumanni* KJ834096 (Li et al. 2016), *Turdus eunomus* NC_028273 (Dong et al. 2018), *Turdus ruficollis* MT712159 (X. Zhang et al. 2021), *Turdus atrogularis* PQ790019 (Jiang et al. 2025), *Turdus pallidus* PQ249424 (direct submission), *Turdus celaenops* LC541445 (Yamamoto et al. 2023), *Turdus kessleri* MG912943 (Song et al. 2018), *Turdus hortulorum* KF926987 (Yan et al. 2016), *Turdus mandarinus* KT601060 (Peng et al. 2016), *Turdus abyssinicus* MT017912 (Behrends et al. 2024), *Turdus migratorius* KJ909198 (Keith Barker 2014), *Turdus rufiventris* KT346357 (Gomes de Sá et al. 2017), *Turdus merula* MN122910 (Margaryan et al. 2021), *Turdus philomelos* KC545406 (Gibb et al. 2015), *Turdus eunomus* NC_028273 (Dong et al. 2018), *Turdus obscurus* MZ424446 (direct submission), *Turdus cardis* NC_046948 (Sun et al. 2020), *Turdus dissimilis* NC_059847 (Gou et al. 2024), *Geokichla sibirica* MK377247 (Sun et al. 2019), *Catharus fuscescens* NC_051013 (Feng et al. 2020), *Catharus ustulatus* CM020378 (direct submission), *Zoothera aurea* KT340629 (Park et al. 2019)*, Myadestes coloratus* PQ872305 (this study), *Myadestes myadestinus* NC_031352 (Anmarkrud and Lifjeld 2017), and *Passer montanus* MW495245 (Lee et al. 2021).

Phylogenetic analysis placed *M. coloratus* as a sister taxon of the only other member of *Myadestes* in the analysis, the extinct *M. myadestinus*, with 100% bootstrap support (Figure 3). Similarly, the genus *Myadestes* was placed as a sister group of the other thrushes (i.e. *Turdus*, *Geokichla*, *Catharus*, and *Zoothera)* with high bootstrap support (Figure 3).

## Discussion and conclusion

*M. coloratus* is a near-threatened bird species (IUCN 2021), which is endemic to eastern Panama and the border with Colombia. However, limited genetic resources are currently available for the species. We report the complete mitochondrial genome sequence of *M. coloratus.* The 16,783 bp mitochondrial genome showed AT content bias, typical of thrushes (Sun et al. 2016; Zhang et al. 2021). It contains 13 protein-coding genes, 22 tRNA genes, two rRNA genes, and a control region. Phylogenetic analysis grouped *M. coloratus* with extinct *M. myadestinus*, confirming its position within *Myadestes* and Turdidae, which is consistent with previous studies using short fragments of mitochondrial genes (Miller et al. 2007).

Our study represents the first complete mitochondrial genome reported for any Neotropical *Myadestes* species and the second of the entire genus, which contains 13 species (Winkler et al. 2020). This mitogenome sequence data provides novel genetic resources for elucidating the evolutionary history of *M. coloratus*, as well as informing the conservation status of this threatened species within its natural habitat. Our findings also highlight the scarcity of molecular data for the genus *Myadestes*. Indeed, despite the genus’ broad distribution, which include the Americas and Hawaii (Bankert et al. 2009), only two species currently have available mitogenomic data. Thus, expanding sequencing efforts to include other underrepresented lineages within the genus will be crucial for future comparative studies across the genus’ range.

## Supplementary Material

Supplementary materials.pdf

## Data Availability

The genome sequence data that support the findings of this study are openly available in GenBank of NCBI at https://www.ncbi.nlm.nih.gov/ under accession no. PQ872305. The associated BioProject, SRA, and BioSample numbers are PRJNA1236001, SRR32695440, and SAMN47378098, respectively.
