# Supplementary material for: Characterization of the mitochondrial genome and phylogenetic position of the Varied Solitaire Myadestes coloratus (Passeriformes: Turdidae)
Source: Mitochondrial DNA B Resour. 2025 Aug 25;10(9):878–83. doi: 10.1080/23802359.2025.2549750 (PMC12381985; doi:10.1080/23802359.2025.2549750)
Supplement: Supplementary materials.pdf [file TMDN_A_2549750_SM7767.pdf]

Supplementary materials

**Characterization of the mitochondrial genome and phylogenetic position of the Varied Solitaire *Myadestes coloratus* (Passeriformes: Turdidae)**

Jorge L. Garzon[a,b*], Celestino Aguilar[c,d*], Kristin Salstontall[b], Carlos F. Arias[b], Catherine B. Viverette[e] and Luis F. De León[b,f]

[a]Integrative Life Sciences Doctoral Program, Virginia Commonwealth University, VA, USA; [b]Smithsonian Tropical Research Institute, Panama, Republic of Panama; [c]Department of Genomics and Proteomics, Gorgas Memorial Institute for Health Studies, Panama, Republic of Panama; [d]Departamento de Microbiologia Humana, Facultad de Medicina, Universidad de Panama, Panama, Republic of Panama; [e] VCU Rice Rivers Center, Virginia Commonwealth University, Richmond, Virginia, USA; [f]Department of Biology, University of Massachusetts Boston, Boston, MA, USA.

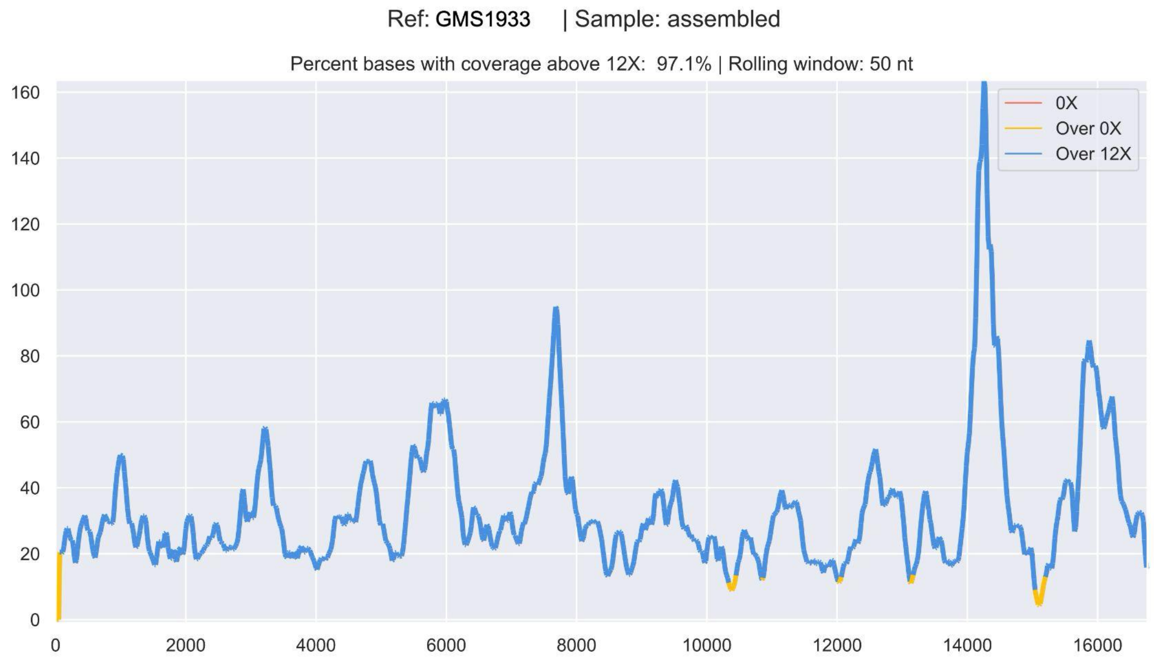


**Supplementary Figure S1.** Coverage depth plot for *Myadestes coloratus* mitochondrial genome. X and Y axis present the nucleotide position of *Myadestes coloratus* mitochondrial genome and coverage depth, respectively.
